# Bioinformatics and Preliminary Functional Analysis of OsPP2C61

**DOI:** 10.3390/genes17040374

**Published:** 2026-03-25

**Authors:** Hao Wang, Enjie Xu, Yujiao Shi, Nuoyan Li, Jinyilin Leng, Yuan Luo, Jianyang Sun, Yaofang Zhang, Zhongyou Pei

**Affiliations:** 1 Tianjin Key Laboratory of Intelligent Breeding of Major Crops, College of Agronomy & Resources and Environment, Tianjin Agricultural University, Tianjin 300384, China; 2 College of Basic Sciences, Tianjin Agricultural University, Tianjin 300384, China

**Keywords:** rice (*Oryza sativa* L.), OsPP2C61, bioinformatics, spatiotemporal expression, subcellular localization

## Abstract

Background: Protein phosphatase 2Cs (PP2Cs) constitutes the largest phosphatase family in plants, playing a pivotal role in signal transduction. Within this family, the PP2C.D subfamily exerts significant influence on cell elongation and stress adaptation by mediating the ‘SAUR-PP2C.D-H+-ATPase’ regulatory module in the auxin signaling pathway. In rice, OsPP2C61 is a PP2C member whose molecular features and potential regulatory context remain unclear. Methods: Our study conducted a preliminary characterization of OsPP2C61 through integrated bioinformatics analysis, spatiotemporal expression profiling, and subcellular localization experiments in tobacco leaf cell. Results: OsPP2C61 encodes a 377-amino-acid protein predicted to be hydrophilic, basic, and structurally unstable. Secondary-structure prediction identified three major elements with random coils as the predominant component, whereas 3D modeling indicated alternating α-helices and β-sheets consistent with a canonical PP2C fold. Phylogenetic inference placed OsPP2C61 within the PP2C.D clade and revealed conserved motifs shared with OsPP2C25, OsPP2C28, and OsPP2C39. Promoter analysis showed enrichment of abscisic acid (ABA)- and methyl jasmonate (MeJA)-responsive elements along with multiple stress-related cis-regulatory motifs. Spatiotemporal expression analysis showed that OsPP2C61 is highly expressed in roots. Subcellular localization assays further demonstrated that the OsPP2C61-GFP fusion protein localizes to the nucleus and the plasma membrane when transiently expressed in epidermal cells of *Nicotiana benthamiana*. Conclusions: This work delivers the first comprehensive characterization of OsPP2C61, establishing a foundation for mechanistic studies and positioning OsPP2C61 as a candidate gene for rice improvement.

## 1. Introduction

Rice (*Oryza sativa* L.) is a major staple crop around the world, with over 50% of the population relying on it as a primary food source [1]. Consequently, understanding the molecular mechanisms underlying rice growth, development, and stress adaptation is essential for improving yield stability and safeguarding national food security. Protein phosphatase 2C (PP2C) constitutes the largest phosphatase family in plants, playing a pivotal regulatory role in multiple signaling pathways involved in developmental and stress responses [2]. Currently, there are 78 identified *PP2C* genes in rice [3], 80 in Arabidopsis [3], 95 in wheat [4], 103 in maize [5], and 131 in Brassica rapa [6].

The PP2C family is commonly classified into 13 subfamilies (A–L) plus an unclassified group, with different subfamilies associated with distinct signaling pathways and biological functions [7]. Among these, the PP2C.D clade has emerged as a key regulator of auxin-mediated “acid growth” during cell elongation. In this regulatory module, SMALL AUXIN UP RNA (SAUR) proteins bind to and inhibit PP2C.D phosphatases, thereby reducing dephosphorylation at the conserved Thr947 residue within the C-terminal autoinhibitory domain of plasma membrane H^+^-ATPases. This inhibition maintains H^+^-ATPases in an activated, phosphorylated state, which promotes apoplastic acidification and facilitates cell expansion [8]. In Arabidopsis, AtSAUR19 can interact with AtPP2C.D2, AtPP2C.D5, and AtPP2C.D6. The *pp2c.d2/d5/d6* mutants located on the plasma membrane exhibit a phenotype of significant elongation of the hypocotyl and inhibition of root growth, similar to overexpression of *SAUR19*-GFP plants, indicating that the PP2C. D subfamily can negatively regulate this process by dephosphorylating key threonine sites of H^+^-ATPase. However, the single mutants *pp2c.d2*, *pp2c.d5*, and *pp2c.d6* showed no significant differences in hypocotyl phenotype, indicating that *PP2C.D2/D5/D6* has a high degree of functional redundancy in regulating cell swelling [9]. In rice, the plasma membrane-localized PP2C.D protein SAL1 negatively regulates H^+^-ATPase activity through threonine dephosphorylation and contributes to aluminum (Al) tolerance by limiting Al^3+^ uptake into root cells [10]. Similarly, OsPP2C36 reportedly interacts with OsA1 (a rice H^+^-ATPase) and modulates root system architecture and drought tolerance through dephosphorylation of the conserved Thr947 residue [11]. The aforementioned findings indicate that members of the PP2C.D subfamily may extensively participate in plant responses to environmental stresses via the ‘SAUR–PP2C.D–H^+^-ATPase’ regulatory module, ultimately influencing key agronomic traits such as plant height, root length, and seed viability. Consequently, the systematic identification and functional characterization of rice *PP2C.D* genes are crucial for deepening our understanding of the regulatory networks governing rice growth, development, and environmental adaptation.

OsPP2C61, a member of the PP2C family identified in our previous yeast two-hybrid screen for proteins interacting with rice SAUR proteins, was classified as belonging to the PP2C.D subfamily based on phylogenetic analysis; however, its precise biological function and regulatory role in rice remain largely unknown. This study aims to preliminarily elucidate the molecular characteristics of PP2C61 through systematic bioinformatics analysis, spatiotemporal expression profiling, and subcellular localization experiments. This work lays the foundation for elucidating its specific role in rice physiological processes and provides potential genetic resources and a theoretical basis for crop genetic improvement.

## 2. Materials and Methods

### 2.1. Plant Materials, Strains, Vectors, and Reagents

Rice (*O. sativa* L.) and *Nicotiana benthamiana* plants were used in this study. The subcellular localization vector employed was pCAMBIA2300. Agrobacterium tumefaciens strain GV3101 competent cells were purchased from Sangon Biotech (Shanghai, China). RNAiso Easy reagent was obtained from Takara Bio (Beijing, China). The reverse transcription kit, high-fidelity DNA polymerase, and seamless cloning kit were purchased from Vazyme (Nanjing, China). Restriction endonucleases BamHI and SalI were acquired from Genesand Biotech (Beijing, China). Quantitative PCR (qPCR) reagents were obtained from Tsingke Biotechnology (Beijing, China). All primer sequences (Table S1) were synthesized, and Sanger sequencing services were provided by Genewiz Biotechnology (Suzhou, China).

### 2.2. Bioinformatic Analysis of Physicochemical Properties and Protein Structure of OsPP2C61

The coding sequence (CDS) and corresponding protein sequence of OsPP2C61 were retrieved from the National Rice Data Center (https://www.ricedata.cn/, accessed on 16 August 2025). Conserved protein domains and family classification were analyzed using the NCBI Conserved Domain Search tool (CD-Search; https://www.ncbi.nlm.nih.gov/Structure/cdd/wrpsb.cgi, accessed on 31 December 2025). Physicochemical parameters (Table S2), including amino acid length, theoretical molecular weight (MW), isoelectric point (pI), instability index, aliphatic index, and grand average of hydropathicity (GRAVY), were predicted using the ExPASy ProtParam online tool (https://web.expasy.org/protparam/, accessed on 31 December 2025). Putative signal peptides and transmembrane helices were predicted using SignalP 5.0 (https://services.healthtech.dtu.dk/services/SignalP-5.0/, accessed on 31 December 2025) and TMHMM 2.0 (https://services.healthtech.dtu.dk/services/TMHMM-2.0/, accessed on 31 December 2025), respectively. Potential Ser/Thr/Tyr phosphorylation sites were predicted using the NetPhos 3.1 server (http://services.healthtech.dtu.dk/service.php?NetPhos-3.1, accessed on 31 December 2025). Secondary structure prediction was performed using the SOPMA tool (https://npsa-prabi.ibcp.fr/NPSA/npsa_sopma.html, accessed on 1 January 2026), and three-dimensional (3D) tertiary structure homology modeling was conducted using SWISS-MODEL (https://swissmodel.expasy.org/interactive, accessed on 1 January 2026) based on available structural templates.

### 2.3. Phylogenetic Analysis and Conserved Motif Identification

Multiple sequence alignment of PP2C protein sequences from rice (*O*. *sativa* L.) and *Arabidopsis thaliana* was conducted using TBtools software (TBtools.v2.452) via the “One Step Build a ML Tree” module (Tables S3 and S4), and a maximum-likelihood (ML) phylogenetic tree was subsequently generated. The resulting phylogenetic tree was visualized and annotated using Evolview v2 (https://www.evolgenius.info/evolview-v2/#login, accessed on 1 January 2026). Conserved motifs within the PP2C family were identified using the Simple MEME (Multiple Em for Motif Elicitation) tool (Table S5), and the top 10 most significant motifs were retained for downstream comparative analysis. The distribution pattern of conserved motifs across different PP2C members was visualized using the “Visualize MEME/MAST Motif Pattern” function integrated in TBtools (TBtools.v2.452).

### 2.4. Promoter Cis-Element Analysis

For each rice PP2C gene, the 2000 bp genomic sequence upstream of the translation start codon (ATG) was extracted from the Rice Genome Annotation Project database and submitted to the PlantCARE database (https://bioinformatics.psb.ugent.be/webtools/plantcare/html/, accessed on 1 January 2026) to identify putative cis-acting regulatory elements using default parameters. The identified cis-element profiles were systematically visualized and annotated using TBtools-II software (TBtools.v2.452) to facilitate comparative promoter analysis (Table S6).

### 2.5. Spatiotemporal Expression Analysis by qRT–PCR

Root, stem, leaf, and panicle tissues were collected from rice plants at the seedling, tillering, jointing, and heading stages, respectively. Total RNA was extracted from each tissue sample using RNAiso Easy reagent and subsequently reverse-transcribed into first-strand cDNA. Quantitative real-time PCR (qRT–PCR) was performed to assess *OsPP2C61* expression levels using *OsACTIN* (*Os03g0718100*) as the internal reference gene (Table S1). The qRT–PCR amplification program was as follows: initial denaturation at 95 °C for 1 min, followed by 40 cycles of denaturation at 95 °C for 10 s and combined annealing/extension at 62 °C for 34 s (two-step cycling protocol). Each sample included three independent biological replicates and three technical replicates.

### 2.6. Subcellular Localization of OsPP2C61

Total RNA was extracted from seedling root tissues of *O. sativa* cv. Nipponbare and reverse-transcribed into cDNA. The full-length coding sequence of OsPP2C61 was amplified using gene-specific primers p2300-OsPP2C61-F/R (Table S1) and inserted into the p2300-GFP expression vector using seamless cloning technology. The resulting recombinant construct (p2300-OsPP2C61-GFP) was verified by Sanger sequencing to confirm sequence fidelity and correct fusion orientation. The validated plasmid was subsequently introduced into GV3101 via electroporation, and transient expression was performed in N. benthamiana leaves using agroinfiltration. Following infiltration, plants were incubated at 25 °C in darkness for 48–72 h to allow protein expression. Green fluorescent protein (GFP) fluorescence signals were observed and recorded using a laser scanning confocal microscope (LSM780; Carl Zeiss Microscopy GmbH, Jena, Germany) to determine the subcellular localization pattern of the OsPP2C61–GFP fusion protein.

## 3. Results

### 3.1. Physicochemical Properties of OsPP2C61

**Figure 1:**
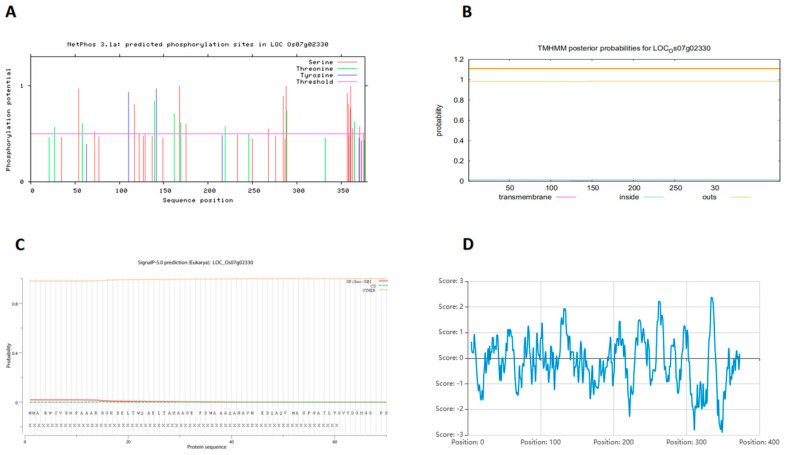
Physicochemical property analyses of OsPP2C61. (**A**) Predicted phosphorylation sites. (**B**) Transmembrane helix prediction. (**C**) Signal peptide prediction. (**D**) Hydropathicity (GRAVY).

The physicochemical properties of OsPP2C61 were systematically analyzed using the ExPASy ProtParam tool. The full-length genomic sequence of rice *OsPP2C61* spans 1460 bp, comprising one intron and two exons; the coding sequence (CDS) extends 1134 bp, encoding 377 amino acids. The relative molecular mass of the OsPP2C61 protein is 40.62 kDa, with a total of 5691 atoms and a theoretical isoelectric point of 8.29, classifying it as an alkaline protein. Its lipophilicity coefficient is 88.62 and its instability coefficient is 45.86, indicating it is an unstable protein. OsPP2C61 contains 47 negatively charged amino acid residues (Asp + Glu) and 49 positively charged residues (Arg + Lys), indicating a net positive charge under physiological pH conditions. NetPhos prediction reveals multiple high-confidence phosphorylation sites, with 17 Ser, 8 Thr, and 2 Tyr residues (Figure 1A), suggesting that post-translational phosphorylation may play a regulatory role in OsPP2C61 function. TMHMM 2.0 analysis predicted no transmembrane helices (Figure 1B), and SignalP 5.0 analysis indicated a very low probability (1.856%) of a signal peptide (Figure 1C), supporting that OsPP2C61 is likely a soluble intracellular protein. The grand average of hydropathicity (GRAVY) value was −0.144, indicating that OsPP2C61 is a hydrophilic protein (Figure 1D).

### 3.2. Structural Features of OsPP2C61

**Figure 2:**
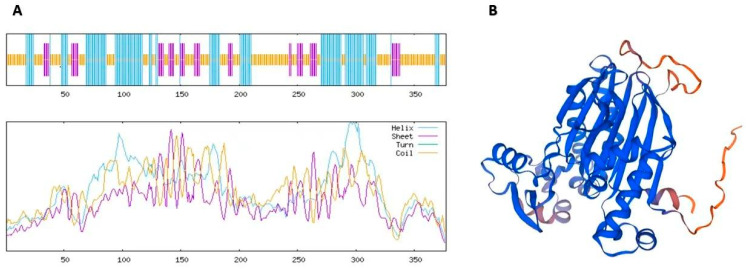
Predicted structure of OsPP2C61. (**A**) Secondary-structure composition. (**B**) Predicted tertiary structure.

The secondary structure of OsPP2C61 was predicted using the SOPMA online tool. Analysis indicates that the OsPP2C61 protein comprises three distinct secondary structural elements: random coils (51.46%), α-helices (34.22%), extended strands (14.32%) (Figure 2A). The predominance of random coils suggests considerable structural flexibility, which may facilitate protein–protein interactions and conformational changes associated with catalytic activity. Tertiary structure modeling of OsPP2C61 was performed using SWISS-MODEL based on homology to known PP2C structures. The predicted three-dimensional structure exhibits an alternating pattern of α-helices and β-sheets: β-sheets form the core scaffold, while peripheral α-helices contribute to overall structural stability; random coils are predominantly distributed across the protein surface, potentially serving as flexible connectors or interaction interfaces. Model quality assessment revealed a global model quality estimate (GMQE) of 0.89, indicating high credibility for the predicted structural model (Figure 2B). Collectively, these structural features are consistent with the conserved architecture of PP2C phosphatases and support the hypothesis that OsPP2C61 functions as a bona fide phosphatase with metal ion-dependent catalytic activity.

### 3.3. Phylogenetic Analysis and Conserved Motif Characterization of PP2C61

**Figure 3:**
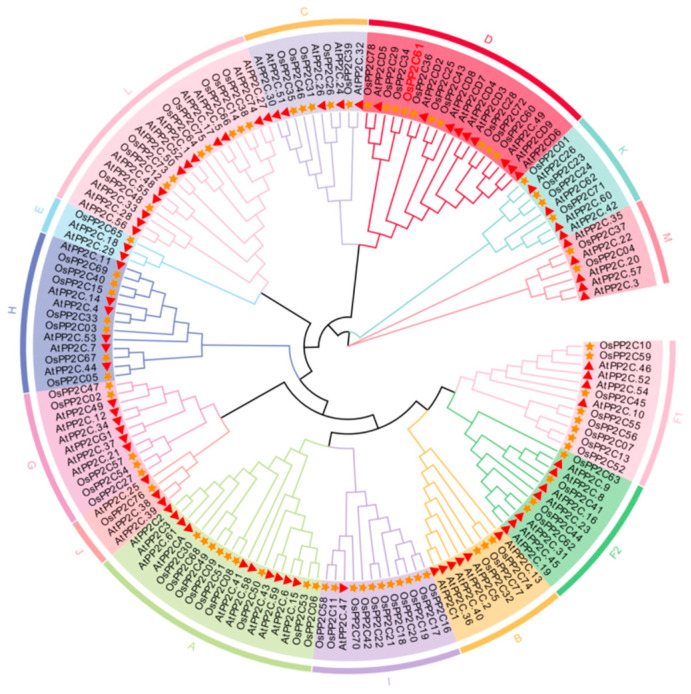
Phylogenetic relationships of *PP2C* family members in Arabidopsis and rice. The outer ring indicates subfamily names, while the inner ring displays specific genes. Red triangles represent Arabidopsis genes, and yellow stars represent rice genes.

**Figure 4:**
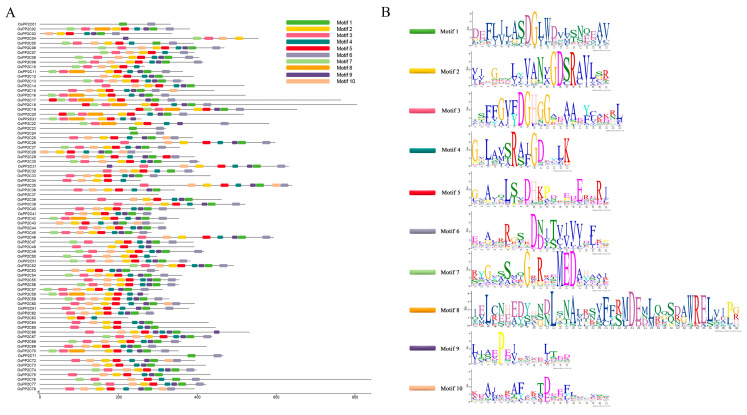
Conserved motif analysis of *OsPP2C61*. (**A**) Motif distribution. (**B**) Motif logo/annotation (as applicable).

To analyze the evolutionary relationships and phylogenetic characteristics of PP2C61, the PP2C protein families in *Arabidopsis thaliana* and *O. sativa* L. were identified using the Phytozome database, the Pfam database, HMMER 3.0 software, and TBtools (TBtools.v2.452). The results revealed that Arabidopsis contains 80 PP2C proteins, while *O. sativa* contains 78 PP2C proteins (Figure 3; Tables S3 and S4). The number of PP2C proteins in Arabidopsis and rice is comparable, indicating that this family has remained relatively conserved throughout plant evolution with no significant gene loss. However, certain differences exist between PP2C proteins across species, suggesting their functional importance may vary among different plants. The phylogenetic tree derived from the analysis classifies PP2C61 within the PP2C.D subfamily. To further elucidate their functions, an analysis of the conserved motifs in all rice PP2C proteins was conducted (Figure 4 and Table S5). The results revealed that ten conserved motifs coexist among members of the *PP2C* gene family, with highly similar motif types, numbers, and positions among members of the same evolutionary branch. For instance, both *OsPP2C34* and *OsPP2C61* contain motifs 2, 3, 4, 5, and 10, with highly consistent distribution patterns, suggesting they may share similar biological functions. Moreover, the vast majority of *PP2C* genes contain motifs 1, 4, and 9 in a conserved sequence order. These three motifs are thus presumed to constitute characteristic sequences of PP2C proteins, playing a crucial role in maintaining protein structure and function. In summary, the variation in the types and number of conserved motifs among *OsPP2C* family members reflects structural diversity, potentially correlating with their diverse biological functions in rice growth and development.

### 3.4. Cis-Element Analysis of the OsPP2C61 Promoter

**Figure 5:**
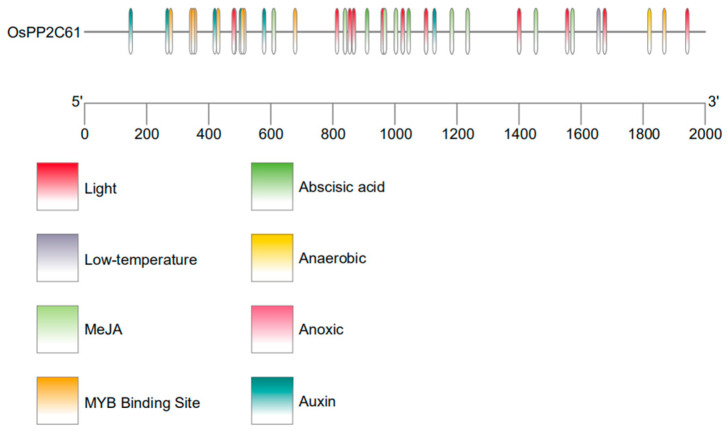
Cis-acting elements in the *OsPP2C61* promoter.

A systematic analysis of cis-acting regulatory elements within the promoter region of *OsPP2C61* was conducted using the PlantCARE database (Figure 5 and Table S6). Multiple hormone-responsive and stress-responsive elements were identified, including abscisic acid response elements (ABREs), auxin response elements, and methyl jasmonate response elements. These in silico predictions suggest that *OsPP2C61* may participate in hormone-regulated signaling pathways.

### 3.5. Organ-Specific Expression Profile of OsPP2C61

**Figure 6:**
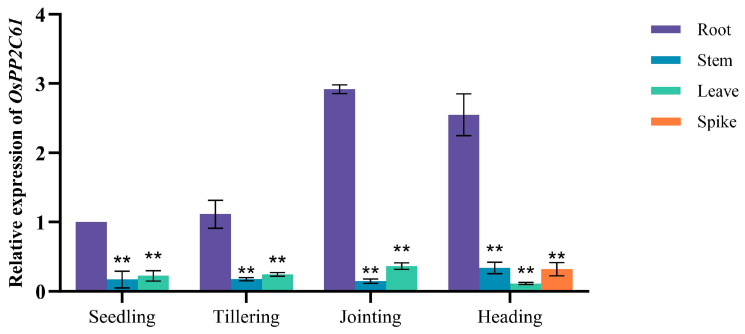
Expression pattern of the *PP2C61* gene in rice. The data represent means ± SE (*n* = 3). ** *p* < 0.01, according to Student’s *t*-test.

The expression profile of OsPP2C61 across different developmental stages and in various tissues was quantified by quantitative real-time PCR (qRT–PCR), using *OsACTIN* (*Os03g0718100*) as the internal reference gene for normalization. Three independent biological replicates and three technical replicates were included for each sample. qRT–PCR analysis revealed that *OsPP2C61* exhibited the highest expression levels in root tissues, with particularly pronounced accumulation at the jointing and heading stages, whereas expression was relatively low in stems, leaves, and panicles across all developmental stages examined (Figure 6). The results show that *OsPP2C61* exhibits elevated expression in roots, particularly during the jointing and heading stages, suggesting potential involvement in root-related processes.

### 3.6. Subcellular Localization of OsPP2C61

**Figure 7:**
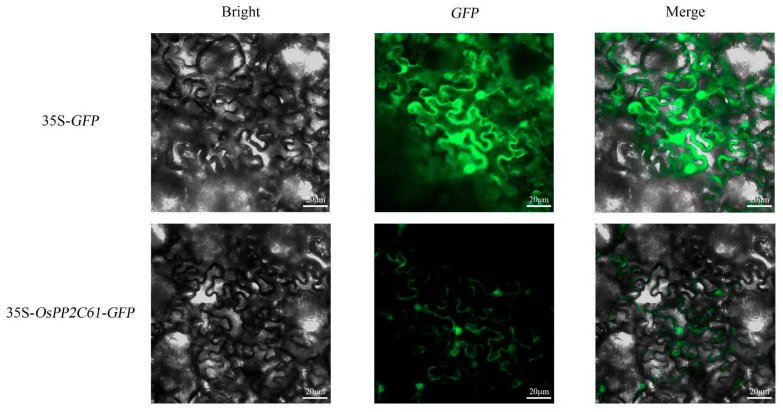
Subcellular localization of *OsPP2C61*–GFP in *Nicotiana benthamiana* leaf epidermal cells, green fluorescent signal indicates the location of GFP protein expression. (Bar = 20 μm).

To determine the subcellular localization of the rice PP2C61 protein (OsPP2C61), this study constructed a pCAMBIA2300-rice *PP2C61*-GFP fusion vector and performed transient expression in tobacco curled epidermal cells via Agrobacterium-mediated infiltration. Confocal laser scanning microscopy revealed that in tobacco leaves infected with the empty vector, fluorescence signals were widely distributed within the nuclei. In contrast, in leaves expressing the *OsPP2C61*–GFP fusion protein, the fluorescence signal was observed predominantly in the nucleus and at the plasma membrane (Figure 7). Notably, because co-localization analyses with known organelle markers were not performed, the precision of this localization result requires further verification.

## 4. Discussion

Our study conducted a phylogenetic analysis by combining PP2C protein sequences from Arabidopsis and *O. sativa* L. A total of 158 PP2C proteins (80 from Arabidopsis and 78 from rice) were classified into 14 monophyletic subfamilies. Compared with previous research [3,12], this study introduces the novel PP2C.M subfamily, which includes the genes *AtPP2C.35*, *AtPP2C.22*, *AtPP2C.20*, *AtPP2C.57*, *AtPP2C.3*, *OsPP2C37*, and *OsPP2C04*. Among these, *AtPP2C.35*, *AtPP2C.3*, and *OsPP2C37* were originally assigned to the *PP2C.A* subfamily; *AtPP2C.22* belonged to the *PP2C.G* subfamily; *AtPP2C.20* was part of the *PP2C.F1* subfamily; and *AtPP2C.57* and *OsPP2C04* were previously ungrouped or orphan genes. The overall classification framework remains highly consistent with previous work, with only minor discrepancies observed at the boundaries of certain subgroups. These variations primarily stem from database updates and advancements in phylogenetic methods, enabling previously ungrouped genes to be assigned to existing subfamilies with higher support values. Based on the phylogenetic results of this study, *PP2C61* is classified within the *PP2C.D* subfamily. Structural predictions further indicate that OsPP2C61 lacks transmembrane domains and signal peptides, suggesting it functions as an intracellular soluble protein. This characteristic aligns with its role in intracellular signal transduction [13].

Previous studies have demonstrated that phosphorylation serves as a crucial molecular switch regulating the enzymatic activity, protein interactions, and subcellular localization of PP2C (protein phosphatase 2C). For instance, in Arabidopsis, the SnRK2 kinase OST1 inhibits the activity of PP2CG1 through phosphorylation, thereby establishing a kinase–phosphatase-positive feedback loop to amplify ABA signaling [14]. RAF family kinase RAF12 phosphorylates HAI2, a member of the PP2C.A subfamily, thereby releasing its inhibition on downstream SnRK2 [15]. In maize, the calcineurin B-like protein-interacting protein kinase ZmCIPK33 phosphorylates Ser60 of the PP2C phosphatase ZmPP2C11, affecting its interaction capacity and enzymatic activity [16]. In the present study, OsPP2C61 was predicted to contain numerous Ser/Thr phosphorylation sites, suggesting that its phosphatase activity and interaction capacity may be modulated through phosphorylation by upstream protein kinases. Such phosphorylation events could directly regulate catalytic activity or indirectly alter binding affinity for potential interacting partners through conformational changes, thereby enabling dynamic regulation of signaling output in response to developmental cues and environmental stimuli.

In silico promoter analysis of *OsPP2C61* identified multiple putative cis-acting elements related to stress and environmental responses, including anaerobic response elements (AREs), low-temperature response elements (LTR), and several light-responsive elements. This suggests its potential involvement in abiotic stress responses, environmental signal perception, and light-mediated developmental regulation. Furthermore, abscisic acid response elements (ABREs) and MYB-binding sites exhibit high enrichment within the promoter region. More importantly, this finding fits with the well-established role of the *PP2C* family in ABA-mediated stress signal transduction [17]. It is indicated that *OsPP2C61* may be regulated by ABA-related signals and MYB-type transcription factors, However, these regulatory relationships are based on bioinformatic predictions and should be validated experimentally.

The “acidic growth” hypothesis proposes that acidification of the plasmolysmic space, mediated by the H^+^-ATPase in the plasma membrane through active proton efflux, promotes cell wall relaxation and accelerates cell elongation; this process is strictly regulated by reversible protein phosphorylation [18]. Previous studies indicate that PP2C.D phosphatase frequently cooperates with SAUR proteins to promote dephosphorylation of H^+^-ATPase at the conserved penultimate threonine residue, thereby reducing proton pump activity and functioning as a molecular ‘brake’ in the auxin signaling pathway [8]. Research in Arabidopsis indicates that the antagonistic interaction between SAUR19 and PP2C.D proteins enhances phosphorylation of the C-terminal domain of the plasma membrane H^+^-ATPase, thereby maintaining its active state and promoting cell expansion via the acid growth mechanism [19]. Conversely, SAUR63 enhances H^+^-ATPase activity by inhibiting PP2C.D5 phosphatase activity, thereby promoting growth in multiple organs [20]. Based on this, it is hypothesized that the rice OsPP2C61 may participate in a functionally similar regulatory module. By interacting with proteins from the rice SAUR family, it could modulate the phosphorylation status of H^+^-ATPase, thereby finely regulating auxin-mediated growth responses.

In this study, qPCR was used to quantitatively assess *PP2C61* expression in roots, stems, leaves, and spikes, and transcript levels were found to be significantly higher in root tissues. However, it should be noted that RNA was extracted from whole-organ homogenates; therefore, the measured mRNA signal represents a mixture derived from dozens of distinct cell types. Accordingly, high expression at the organ level may reflect either broad expression across multiple cell types or highly restricted expression in only a few specific cell populations. Thus, the data presented here represent only organ-averaged transcript abundance normalized to the Actin reference gene and lack spatial resolution. To precisely define the expression domains of *PP2C61* in root tissues and clarify their association with specific cell types and developmental gradients, future studies should employ spatial cell biology approaches, such as spatial transcriptomics or single-cell in situ analysis, to further characterize *PP2C61* expression at cellular resolution, thereby providing more compelling spatial molecular evidence for its functional mechanisms [21].

In the broader context of plant phosphatase biology, members of the *PP2C* family have been shown to participate in multiple signaling pathways related to development and stress responses, including ABA signal transduction, osmotic stress adaptation, pathogen defense responses, and auxin-mediated cell elongation [22]. *OsPP2C61* is highly expressed in roots, and its fusion protein was detected in both the nucleus and the plasma membrane in a tobacco heterologous expression system, suggesting that this protein may function in multiple subcellular compartments. It should be noted that the subcellular localization assay was conducted under dark-culture conditions. Although this condition was experimentally required, it may affect cellular physiology and thereby alter protein localization patterns. In future work, localization validation in rice protoplasts could be performed to further confirm the accuracy of these results.

Currently, the precise biochemical function of the *OsPP2C61* gene and its upstream regulators and downstream substrates, as well as its specific physiological role in abiotic stress responses such as drought and salinity, remain unknown. Based on preliminary clues from this study, including its promoter enrichment with stress-responsive elements and high expression in roots, subsequent research should investigate the dynamic changes in the OsPP2C61 protein during short-term stress responses (1–3 h) and long-term adaptation phases (24–48 h) at cell-type-specific resolution. Building on this foundation, techniques such as yeast two-hybrid and co-immunoprecipitation coupled with mass spectrometry (IP-MS) should be employed to identify its interacting protein network. Finally, by constructing genetically edited or overexpressing rice materials, the in vivo functional mechanisms and contributions of *OsPP2C61* in rice growth, development, and adaptation to field environments should be systematically elucidated.

## 5. Conclusions

This study analyzed the sequence characteristics, evolutionary relationships, promoter cis-elements, organ-specific expression profile, and subcellular localization of OsPP2C61. Results indicate that *OsPP2C61* exhibits high expression in rice roots, and *PP2C61*-GFP is widely distributed in the nucleus and cell membrane. It should be noted that the expression profile reflects the average mRNA level across mixed cell populations, which may encompass at least ten different cell types, each with distinct mRNA abundance and dynamics. Future investigations focusing on cell type-specific expression patterns in the spatial context will be essential to fully elucidate the functional roles of *OsPP2C61*. These findings collectively establish foundational resources for its further functional characterization.

## Data Availability

The original contributions presented in this study are included in the article/Supplementary Materials. Further inquiries can be directed to the corresponding authors.

## Supplementary Materials

The following supporting information can be downloaded at https://www.mdpi.com/article/10.3390/genes17040374/s1: Table S1: Sequence of primers used for this study. Table S2: General information of identified OsPP2C61 gene in *Oryza sativa* L. Table S3: *Arabidopsis thaliana* PP2C gene family. Table S4: *Oryza sativa* L. PP2C gene family. Table S5: Conserved amino acid motifs and annotation of OsPP2Cs. Table S6. Cis-elements analysis of *OsPP2C61* gene.

## Abbreviations

The following abbreviations are used in this manuscript:

PP2C	Protein phosphatase 2C
SAUR	SMALL AUXIN UP RNA
ABA	Abscisic acid
MeJA	Methyl jasmonate
Al	Aluminum
CDS	Coding sequence
MW	Molecular weight
pI	Isoelectric point
GRAVY	Grand average of hydropathicity
3D	Three-dimensional
GFP	Green fluorescent protein
GMQE	Global model quality estimation
ARE	Anaerobic induction element
LTR	Low-temperature responsive element
MBS	MYB-binding site
ABRE	ABA-responsive element
RNAi	RNA interference
